# Association of Serotonergic Pathway Gene Polymorphisms With Behavioral Parameters in Patients With Opioid Dependence

**DOI:** 10.7759/cureus.19310

**Published:** 2021-11-06

**Authors:** Siddharth Sarkar, Renu Singh, Arundhati Sharma, Muzaffar A Pandit, Ranjan Gupta, Deepika Singhal, Raka Jain, Yatan P Balhara

**Affiliations:** 1 National Drug Dependence Treatment Centre, All India Institute of Medical Sciences, New Delhi, IND; 2 Department of Anatomy, All India Institute of Medical Sciences, New Delhi, IND

**Keywords:** impulsiveness, pcr, polymorphism, serotonin, opioid dependence

## Abstract

Background and aims

Opioid dependence is a chronic, relapsing substance use disorder with a multifactorial etiology, including a genetic component. Serotonin pathway gene polymorphisms have been an important focus of research for psychiatric disorders, including substance use disorders. This study aimed to identify the association of serotonin pathway gene polymorphisms with self-harm, depressive symptoms, impulsiveness, and aggression in patients with opioid dependence.

Method

The study group comprised 366 subjects with opioid dependence and 200 healthy volunteers. Patients were assessed for a history of self-harm, depressive symptoms, impulsiveness, and aggression using standard tools. Genomic DNA was used for genotyping of four polymorphisms - 5-HTTLPR, STin2 VNTR, *TPH1* A218C, and *TPH2 *G703T.

Results

The short allele of 5-HTTLPR polymorphism showed a significant difference between cases (59.8%) and controls (40.1%) (*p*=0.001) and revealed an association with age at opioid dependence (*p*=0.033). There was a borderline significance of the short allele of 5-HTTLPR with the duration of opioid use (*p*=0.061) and non-plan impulsivity (*p*=0.076), suggesting a role of 5-HTTLPR in the susceptibility of opioid dependence. The other markers did not differ between cases and controls. However, the STin2A polymorphism revealed a significant association with anger scores, which may indicate its role in aggressive behavior.

Conclusions

The present study, the first of its kind, suggests an association of 5-HTTLPR polymorphism with opioid dependence and STin2A polymorphism with aggressive behavior among opioid-dependence patients, signifying the role of these markers in our patient population.

## Introduction

Substance use disorders are among the more common psychiatric disorders worldwide [1]. Globally, about 652,000 people aged 12 years or older have had heroin use disorder, and almost one-fourth of the people who use heroin become addicted. The death rate due to heroin consumption in the US increased from 5,925 in 2012 to 8,257 in 2013 [2]. In India, the prevalence of opioids, cannabis, and alcohol is 0.7%, 3.0%, and 21.4%, respectively [3]. Among the opioid users, 22.3% are dependent users [3]. A recent survey in India published in 2019 has shown that the absolute number of individuals dependent on opioids in India has increased by more than five times over a period of 15 years [3-4]. Substance use disorders are associated with a considerable burden on the healthcare system [5]. Several studies have implicated the role of substance use in the occurrence of self-harm behaviors, depressive symptoms, impulsiveness, and aggression [6-10].

Studies have reported on the association of polymorphisms of serotonergic pathway genes in substance use disorders [11-12]. Serotonin transporter (Solute Carrier Family 6 Member 4 [SLC6A4]/5-HTT) is a sodium-dependent transporter localized at the presynaptic neuronal membranes, involved in the recycling of serotonin between the synapse and the neuronal cell. The serotonin transporter-linked promoter region (5-HTTLPR) and STin2 Variable Number Tandem Repeat (VNTR) polymorphisms of the SLC6A4 gene have been reported to be associated with the risk of developing substance use disorders, as these polymorphisms are known to regulate the transcriptional activity of the SLC6A4 gene [13-14]. Serotonin is a crucial neurotransmitter that has a significant role in mood regulation, and low serotonin levels in the brain are associated with depression and suicidal attempts. Variants in the serotonin transporter (SLC6A4), i.e. rs25532, 5-HTTLPR, and STin2 VNTR, are associated with major depression, schizophrenia, and bipolar disorders. 5-HTTLPR polymorphism, present within the promoter region of the SLC6A4 gene, is known to affect its transcriptional activity. The deletion variant or short allele is known to reduce the transcriptional activity of the gene [13]. Studies have also revealed an association of 5-HTTLPR with depression and suicide attempts [15]. STin2 VNTR involves two major alleles (STin2.10 and STin2.12), i.e. 10 and 12 repeats [16]. The biological function of this polymorphism is not yet clear, but STin2.12 has been reported to possess greater transcriptional activity than STin2.10 [17].

Tryptophan hydroxylase (TPH) catalyzes the rate-limiting step in the serotonin (5-HT) synthesis. There are two TPH genes, TPH1 and TPH2, expressed in the central nervous system involved in serotonin metabolism. Studies indicate 5-HT synthesis through TPH2 influences nervous tissues, whereas TPH1 is responsible for the synthesis and action of 5-HT in peripheral organs. Partial impairment of brain 5-HT synthesis caused by polymorphism rs4570625 (G703T) of the gene encoding TPH2 has been linked to several psychiatric phenomena like obsessive-compulsive disorder, increased aggressiveness, and mood and anxiety disorders, leading to serious conditions like suicidal risks [18-20]. A study reports alteration in anxiety-like behavior in TPH2-deficient mice, which is accompanied by adaptation changes of 5-HT(1A) receptors and the associated signaling pathway [21].

The present study aimed to assess the association of serotonin pathway gene polymorphisms with self-harm, depressive symptoms, impulsiveness, and aggression in patients of opioid dependence in the north Indian population.

## Materials and methods

Setting and participants

The study was in accordance with the declaration of Helsinki and was approved by the institutional ethics committee of the All India Institute of Medical Sciences, New Delhi (IEC-468/07.10.2016, RP-17/2016). The study was conducted at a public-funded addiction treatment center in north India, which is affiliated with a medical school. The center treats patients with a variety of substance use disorders and offers both inpatient and outpatient care facilities. Opioids are the most common substance of use for which treatment is being provided at the center. Treatment is provided in the form of detoxification or opioid substitution treatment based upon clinical considerations and patient preference.

The inclusion criteria (for cases) were males with a diagnosis of opioid dependence (as per International Classification of Diseases (ICD 10) diagnostic criteria), willing to participate in the study, and were not in active withdrawal (reflected by Objective Opioid Withdrawal Scale scores) [22]. Females were excluded, as they comprise about only 2% of the treatment-seeking population at the center. The exclusion criteria were substance dependence (other than opioid/tobacco), severe medical or psychiatric illness, or those who had a current diagnosis of psychotic or mood spectrum disorder as per Mini-International Neuropsychiatric Interview [23] were excluded from the study. The interviews were conducted by trained psychiatrists. We expected depressive symptoms, anxiety symptoms, impulsiveness, and aggression to be present in this population, though we excluded patients with a diagnosable psychotic illness, bipolar disorder, or major depressive episode.

The demographic profile of subjects and controls was noted using a structured questionnaire and the patients with opioid dependence were also interviewed using Hamilton Depression Rating Scale [24], Barratt’s Impulsiveness Scale [25], and Buss Perry Aggression Questionnaire [26].

Genetic analysis

A detailed family history of alcohol use was recorded, and five milliliters of venous blood were drawn in ethylenediaminetetraacetic acid (EDTA) vials from all the participants. Genomic DNA was isolated using the standard salting-out method [27] and processed for polymerase chain reaction (PCR) amplification to screen the polymorphisms 5-HTTLPR, STin2 VNTR, and TPH2(G703T) from the serotonin pathway using the primers and restriction enzymes described previously [28-29]. The details of the same are presented in the Appendix.

Instruments

Barratt’s Impulsiveness Scale [25]

This is a 30-item instrument for the assessment of impulsiveness. Each of the items is rated on a four-point Likert scale from “rarely/never” to “always/almost always.” The scale has six first-order factors and three second-order factors. The total score of Barratt’s Impulsiveness Scale has been found to be an internally consistent measure of impulsiveness and has potential clinical utility for measuring impulsiveness.

Buss Perry Aggression Questionnaire [26]

This is a 29-item instrument to assess aggression. Each of the items is rated on a Likert-rated scale from “extremely uncharacteristic of me” to “extremely characteristic of me.” The scale has been divided into four factors, namely verbal aggression, physical aggression, anger, and hostility. The scale has good internal consistency and reliability over time. Men score higher on physical aggression, verbal aggression, and hostility as compared to women.

Statistical analysis

The distribution of the quantitative variables as parametric or nonparametric was determined by the Shapiro-Wilk test. Genotype and allele frequencies were compared between subjects using Pearson’s chi-square test. Clinical parameters were compared between opioid-dependent individuals with self-harm and those without such harm using Pearson’s chi-square test or student's t-test. Correlation of genotypes with factors like age, age at first opioid use, duration of opioid use, age at opioid dependence, Buss Perry, and Barratt Aggression scores was done using the Kruskal-Wallis test. Analysis was done using SPSS version 21 software (IBM Corp. Armonk, NY). Missing value imputation was not required, and the p-value for the comparisons was set at 0.05.

## Results

A total of 437 patients with opioid dependence were recruited for the study, of which 366 were included in the analysis. Others were excluded as either the sample collected was too little for the genetic analysis or they were currently dependent on other substances as well (apart from tobacco). The characteristics of the patients included in the study are presented in Table 1. All the patients with opioid dependence were males, and the mean age was 33.9 (±12.5). Current dependent use of tobacco was present in about three-fifths of the population. The sample comprised predominantly heroin users, though a substantial proportion comprised of raw opium/poppy husk users as well. About six percent of the sample was injecting drug users. A total of 200 controls were recruited.

**Table 1:** Demographic characteristics of opioid dependence patients and behavioral parameters *p < 0.05; Mean (±Standard deviation) or frequency (percentage), †Those with current dependent use were excluded

Variable	Overall (n = 366)	With a history of self-harm or suicide attempt (n = 61)	Without a history of self-harm or suicide attempt (n = 305)	Comparison (p-value)
Age in years	33.9 (±12.5)	27.1 (±9.2)	35.3 (±12.7)	t = 5.876 (<0.001)*
Married currently	215 (58.7)	25 (41.0)	190 (62.1)	χ^2^= 8.667 (0.003)*
Currently employed	306 (83.1)	50 (82.0)	254 (83.0)	χ^2^= 0.062 (0.803)
Education up to high school	290 (79.2)	51 (83.6)	239(78.4)	χ^2^= 0.561 (0.454)
Urban residence	208 (56.8)	44 (72.1)	164 (53.6)	χ^2^= 6.985 (0.008)*
Heroin user	226 (61.7)	55 (90.2)	166 (54.4)	χ^2^=25.666 (<0.001)*
Injecting drug use	22 (6.0)	3 (4.9)	19 (6.2)	χ^2^= 0.010 (0.922)
Barratt Impulsiveness Scale				
Attentional	15.9 (±2.3)	17.2 (±2.2)	15.7 (±2.2)	t = 4.789 (<0.001)*
Motor	20.6 (±2.8)	20.7 (±2.7)	20.6 (±2.8)	t = 0.297 (0.763)
Non-plan	27.0 (±3.6)	28.7 (±3.4)	26.7 (±3.5)	t = 4.199 (<0.001)*
Buss Perry Aggression Questionnaire				
Physical	23.0 (±6.2)	25.7 (±5.8)	22.4 (±6.1)	t = 3.850 (<0.001)*
Verbal	11.8 (±4.3)	13.6 (±4.3)	11.4 (±4.3)	t = 3.646 (<0.001)*
Anger	18.5 (±4.6)	20.5 (±3.6)	18.1 (±4.6)	t = 4.414 (<0.001)*
Hostility	16.1 (±5.1)	18.4 (±4.7)	15.7 (±5.1)	t = 3.832 (<0.001)*
Hamilton Depression Rating Scale	2.2 (±2.2)	3.7 (±2.2)	2.0 (±2.1)	t = -5.784 (<0.001)*

The parameters on the Hamilton Depression Rating Scale, Barratt Impulsiveness Scale, and Buss Perry Aggression Questionnaire are presented in Table 1. It was seen that 61 participants, i.e., 16.7% of the sample had a history of self-harm or suicide attempts. Among these, 38 participants had a history of self-harm without intent to die, and 35 participants had a history of suicide attempts with intent to die. This reflects that 12 participants had a history of suicide attempts with intent to die and self-harm without intent on different occasions.

The genotype and allele frequencies of the 5’HTTLPR, STin2 VNTR, TPH1(A218C), and TPH2(G703T) polymorphisms are shown in Table 2. There was no deviation from Hardy Weinberg Equilibrium for 5’HTTLPR, TPH1, and TPH2. There was a significant difference of 5-HTTLPR in the genotype and allele frequencies between opioid-dependent cases and controls while other polymorphisms like STin2 VNTR, TPH1 (A218C), and TPH2(G703T) did not show any difference.

**Table 2:** Genotype and allele frequencies in patients with opioid dependence (n = 366) *Significant at p < 0.05

Marker	Genotypic distribution (frequencies)			p-value	Allelic distribution (frequencies)		p-value
5-HTTLPR	S/S	S/L	L/L	<0.001*	S	L	0.001*
cases	137 (37.4%)	164 (44.8%)	65 (17.8%)		438 (59.8%)	294 (40.1%)	
controls	43 (22.0%)	115 (57.0%)	42 (21.0%)		201 (50.2%)	199 (49.7%)	
STin2 VNTR	10/10	10/12	12/12	0.935	10	12	0.690
cases	80 (21.9%)	127 (34.7%)	159 (43.4%)		287 (39.2%)	445 (60%)	
controls	42 (21.0%)	68 (34.0%)	90 (45.0%)		152 (38%)	248 (62%)	
TPH1	C/C	C/T	T/T	0.493	C	T	0.324
cases	179 (48.9%)	141 (38.5%)	46 (12.5%)		499 (68.2%)	233 (31.8%)	
controls	108 (54.0%)	68 (34.0%)	24 (12.0%)		284 (71.4%)	116 (28.5%)	
TPH2	G/G	G/T	T/T	0.475	G	T	0.243
cases	193 (52.7%)	147 (40.2%)	26 (7.1%)		533 (72.8%)	199 (27.2%)	
controls	114 (57.0%)	76 (38.0%)	10 (5.0%)		304 (76.0%)	96 (24.0%)	

The short allele of 5-HTTLPR polymorphism showed a significantly higher frequency in cases (59.8%) compared to controls (40.1%) (p=0.001). It was found that age at opioid dependence in cases with 5-HTTLPR was higher with SS (short allele) 24.00(20.00-30.00) as compared to LL (long allele) 22.00(19.00-27.00) and SL 21.0(18.00-26.00) genotypes (p=0.033). Further, the pair-wise comparison revealed the 5-HTTLPR SS genotype to be associated with higher opioid age of dependence as compared to SL genotypes (p=0.012) while 5-HTTLPR also showed an association with the duration of opioid use (p=0.061) and the Barratt non-plan impulsivity score (p=0.076) but did not reach statistical significance (Table 3).

**Table 3:** Association of marker genotypes with opioid use parameters The values in each cell are represented as median (Interquartile range). *Significant at 5% level

Markers with genotype		Age at First Use in years	Age at Dependent Use in years	Duration of Use in years	Buss Perry Aggression Questionnaire				Barratt Impulsiveness Scale			Hamilton Depression Rating Scale
					Physical	Verbal	Anger	Hostility	Attentional	Motor	Non plan	
5’-HTTLPR	S/S	23.00 (19.00-29.00)	24.00 (20.00-30.00)	4.00 (2.00-8.00)	22.00 (18.00-28.50)	11.00(8.00-16.00)	19.50 (15.00-22.00)	16.00 (11.00-20.00)	16.00 (14.00-18.00)	21.00 (19.00-23.00)	27.00 (25.00-30.00)	1.00(0.00-4.00)
	S/L	20.00 (17.00-26.00)	21.00(18.00-26.00)	3.00 (2.00-5.00)	22.00 (17.00-28.00)	12.00 (8.00-16.00)	19.00 (15.00-22.00)	16.00 (12.00-20.00)	16.00 (14.00-17.00)	21.00 (19.00-22.00)	26.00 (24.00-29.00)	2.00(0.00-4.00)
	L/L	21.00 (17.00-26.25)	22.00 (19.00-27.00)	3.00 (1.00-6.00)	24.00 (18.00-28.00)	10.00 (8.00-13.50)	19.00 (14.00-22.00)	15.00 (11.00-20.00)	16.00 (14.00-17.50)	20.00 (18.00-22.00)	27.00 (25.00-30.00)	2.00(0.50-4.00)
	p-value	0.104	0.033*	0.061	0.801	0.282	0.906	0.900	0.759	0.235	0.076	0.292
STin2 VNTR	10/10	21.50 (18.00-26.75)	22.00 (19.00-27.00)	3.5 (1.00-6.00)	24.00 (18.25-29.00)	12.00 (8.00-16.00)	20.50 (16.00-23.00)	17.00 (13.00-20.75)	16.00 (14.00-17.00)	21.00 (19.00-22.00)	26.00 (24.00-28.75)	2.00(0.00-4.00)
	10/12	21.00 (17.00-29.00)	22.00 (18.00-30.00)	4.00 (2.00-7.00)	23.00 (17.00-29.00)	11.00 (8.00-16.00)	18.00 (14.75-22.00)	15.00 (12.00-19.00)	16.00 (14.00-17.00)	20.00 (19.00-22.00)	27.00 (25.00-30.00)	2.00(0.00-3.00)
	12/12	21.00 (18.00-26.00)	23.00 (19.00-26.50)	3.00 (2.00-5.00)	22.00 (18.00-26.00)	12.00 (8.00-15.00)	19.00 (15.00-22.00)	16.00 (11.00-20.00)	16.00 (14.00-18.00)	21.00 (19.00-22.00)	27.00 (24.00-29.00)	2.00 (0.00-4.00)
	p-value	0.836	0.752	0.503	0.320	0.624	0.034*	0.525	0.668	0.471	0.592	0.464
TPH1 A218C	A/A	22.00 (19.00-27.00)	23.00 (19.00-27.00)	4.00 (2.00-7.00)	22.00 (18.00-28.00)	11.00 (8.25-16.00)	19.00 (15.00-22.00)	17.00 (13.00-20.00)	16.00 (14.25-18.00)	21.00 (19.00-22.00)	27.00 (25.00-29.75)	2.00 (0.00-4.00)
	A/C	20.00 (18.00-27.00)	22.00 (19.00-27.00)	3.00 (1.00-5.00)	23.50 (18.00-28.00)	12.00 (8.00-15.00)	19.00 (15.00-22.00)	16.00 (11.00-20.00)	16.00 (14.00-17.00)	21.00 (19.00-22.00)	26.00 (24.00-29.00)	2.00 (0.00-4.00)
	C/C	20.00 (17.00-29.25)	21.00 (18.00-33.00)	3.00 (2.00-5.25)	23.00 (16.00-28.00)	10.00 (7.00-13.00)	18.00 (13.50-21.50)	14.00 (10.00-19.00)	16.00 (14.00-17.00)	20.00 (18.00-22.00)	26.00 (24.00-29.00)	1.00 (0.00-3.50)
	p-value	0.488	0.584	0.053	0.669	0.051	0.197	0.116	0.236	0.597	0.341	0.681
TPH2 G703T	G/G	21.00 (18.00-26.00)	22.00 (19.00-27.00)	3.00 (2.00-6.00)	23.00 (18.00-28.00)	12.00 (8.00-16.00)	19.00 (15.00-22.00)	16.00 (11.00-20.00)	16.00 (14.00-18.00)	20.00 (19.00-22.00)	27.00 (24.00-29.00)	2.00 (0.00-4.00)
	G/T	22.00 (19.00-28.25)	23.00 (20.00-29.00)	3.00 (2.00-5.00)	22.00 (18.00-28.00)	11.00 (8.00-15.00)	19.00 (15.00-22.00)	16.00 (12.00-19.00)	16.00 (14.00-17.00)	21.00 (19.00-22.00)	27.00 (25.00-29.00)	2.00 (0.00-4.00)
	T/T	20.00 (17.00-23.75)	21.00 (18.25-24.00)	5.00 (3.00-9.50)	23.50 (18.00-29.50)	13.00 (11.00-16.00)	20.50 (17.75-23.00)	18.00 (13.00-20.00)	16.00 (14.00-17.00)	21.00 (19.00-23.00)	26.00 (24.00-30.00)	2.00 (0.00-5.00)
	p-value	0.281	0.287	0.193	0.672	0.318	0.248	0.588	0.553	0.492	0.696	0.742

STin2A polymorphism revealed an association with Buss Perry anger scores, which showed the 10/10 repeat genotype to be associated with median scores of 20.00 (interquartile range [IQR] 16.00-23.00) compared to scores of 10/12 repeats (median 18.00, IQR 14.75-22.00) and 12/12 repeats (median 19.00, IQR15.00-22.00) (p=0.034). Further, the pair-wise comparison revealed STin2A ‘10/10’ repeats to be associated with higher Buss Perry anger score as compared to ‘10/12’ repeat genotypes (p=0.009) (Table 3).

Additionally, the relationship of self-harm with clinical data is presented in Table 1. It was seen that those individuals with a history of self-harm or suicide attempt were significantly younger than those who had not attempted. Also, they were less likely to be married, have urban residence, and be a heroin user. It was seen that those with a history of self-harm or suicide attempt were more likely to have more attentional and non-plan impulsivity, and have a greater extent of physical, verbal, anger, and hostility aggression.

## Discussion

The present study reports on the association of 5HTTLPR, STin2, TPH1 (A218T), and TPH2 (G703T) polymorphisms of the serotonergic pathway with self-harm, depressive symptoms, impulsiveness, and aggression in opioid dependence. Results revealed an association of short allele of 5-HTTLPR polymorphism with opioid dependence and with the age of opioid dependence. The 10/10 repeat genotype of STin2A polymorphism has shown significant association with the Buss Perry anger score in opioid dependence, which may suggest a role of this polymorphism in aggressiveness. Demographically, the sample of the study consisted exclusively of males (females were excluded, as they comprised less than 2% of the treatment-seeking population at the center and we wanted to homogenize the sample). Though the main opioid being used by the study population was heroin, a considerable proportion of the participants were using natural opiates (raw opium and poppy husk). This pattern of use has been echoed in other clinic-based studies from India [30], but is quite different from the pattern of opioid consumption in the treatment-seeking population in other parts of the world [31-32].

The present study revealed the short allele of 5-HTTLPR has a significantly higher frequency in patients with opioid dependence compared to controls. Studies by Gerra et al. [33] and Wang et al. [34] also reported a significant association of 5-HTTLPR with opioid dependence, which is in concordance with our findings. According to a meta-analysis by Lin and Wu [35], the ‘S’ allele has been found to be more common in the opioid-dependent population. The ‘S’ allele has reduced transcriptional activity and is associated with impulsive behavior, suicidal attempts, and violent behavior. The present study also revealed the 5-HTTLPR ‘S’ allele to be associated with a higher age of opioid dependence (p=0.012), duration of opioid use (p=0.061), and non-plan impulsivity (p=0.076). The results of the present study suggest the ‘S’ allele of 5-HTTLPR polymorphism is associated with delayed use of opioids. Regular and problematic opioid use may occur much later though experimental use may occur initially due to impulsive behaviors. This suggests that the later age of onset of dependence (even with no significant difference in onset of use) may reflect the instability of the course of opioid use disorder before it becomes entrenched as regular dysfunctional use in the form of dependence. These findings are in concurrence with other studies on alcohol dependence, which reported the association of the 5-HTTLPR ‘S’ allele with age, duration of use, gene interaction, and type of substance abuse [12,36-37].

Another marker studied is STin2 VNTR, present in intron 2 of the SLC6A4 gene. Though the frequency of STin2 VNTR in cases and controls was similar in the present study, which is also in concordance with previous studies, the 10/10 genotype shows a significant association with the Buss Perry anger score. Some studies have hypothesized the role of serotonin deficiency in human aggression and have identified reduced serotonergic tone associated with increased risk for aggression [38-40]. In 2018, Hemmings et al. also report STin2 variants as an important regulator of aggression [41]. Therefore, the present study validates the previous reports suggesting an association of STin2 10/10 genotype with aggressiveness in opioid dependence.

The other two markers screened in the present study are A218C and G703T of the TPH1 and TPH2 genes, respectively. The polymorphism A218C is located at a potential GATA transcription factor-binding site and is reported to affect the expression of the TPH1 gene [42]. Studies have revealed the association of TPH1 A218C with impulsivity, suicidality [43], and opiate dependence [44]. TPH2 G703T has been linked to psychiatric disorders such as alcohol dependence, schizophrenia, and major depression [18-20], leading to serious conditions like suicidal risks. However, in the present study, no such association of A218C and G703T polymorphisms was identified with opioid dependence.

Other findings of the study were that suicide attempts and self-harm behaviors in patients with opioid dependence were associated with clinical predictors like younger age, being unmarried, having an urban residence, and being a heroin user. Furthermore, opioid dependence patients with a suicide attempt history were found to have high rates of impulsiveness and aggression when compared to opioid dependence patients without such a history. The results are in concordance with previous studies, which suggested a relationship between impulsiveness and self-harm behavior among young adults and adolescents [45-46]. This is possibly contributed by risky decision-making and deficits in inhibitory control or may be due to undiagnosed attention deficit hyperactivity disorder (ADHD). Aggression has also been associated with self-harm attempts in general. There is evidence to support that aggression and anger are linked to suicidal attempts in substance-using populations [47-48].

The strengths of the present study lie in having a fairly large sample size, using Indian ethnicity, which has not been frequently reported in the published literature, and looking at clinical determinants like impulsiveness and aggression in addition to the serotonin pathway gene polymorphisms. The limitations of the study include a single-center sample from northern India rather than using multiple sites, a clinic-based sample rather than a community sample, and assessing only four genetic polymorphisms. Assessment of self-harm and suicide attempt, impulsiveness, and aggression was based upon self-report from the patients. The lifetime history of suicide or self-harm attempt was considered instead of imposing a time frame, and it does not preclude the likelihood of an attempt in the future.

## Conclusions

To the best of our knowledge, this is the first report on the association of 5HTTLPR, STin2, TPH1 (A218T), and TPH2 (G703T) polymorphisms in opioid dependence in the Indian population, in which the SLC6A4 5HTTLPR and STin2 VNTR have shown an association with opioid dependence and aggressiveness respectively among the patients. The study of these markers may help in a better understanding of the neurobiology of opioid dependence and its relation with aggression and impulsiveness. Impulsiveness and aggression are common phenotypic expressions associated with opioid dependence and in those with self-harm attempts. Understanding which risk alleles relate to increased chances of aggression and impulsivity may lead to more focussed attempts to address these using psychotherapeutic, and possibly pharmacological means.

## Appendices

Genetic analysis

A reaction mixture of 25 μl was prepared using 200 ng genomic DNA, specific primers (0.5 μM each), MgCl2 (1.5 mM), deoxyribonucleotide triphosphates (dNTPs; 0.2 mM), 1× PCR buffer (Thermofisher Scientific, Waltham, Massachusetts), and Taq polymerase (0.5 U; Thermofisher Scientific) in a thermocycler (ABI 9700, [ABI], Foster City, CA). PCR cycling conditions were 7 min initial denaturation at 95 °C, followed by 35 cycles of 94 °C for 30 s, 55 °C-64 °C for 1 min, 72 °C for 1 min, and a final extension at 72 °C for 10 min. The amplified products were genotyped using PCR-restriction fragment length polymorphism (RFLP), and the alleles were scored. One marker TPH1(A218C) was screened using the TaqMan Genotyping assay using primers designed by the manufacturer (Thermofisher Scientific) and TaqMan Universal PCR Master Mix in Step One plus thermocycler using standard conditions (Applied Biosystems, Waltham, Massachusetts).
